# The lived experiences of healthcare during pregnancy, birth, and three months after in women with type 1 diabetes mellitus

**DOI:** 10.1080/17482631.2019.1698496

**Published:** 2019-12-11

**Authors:** Helena Dahlberg, Marie Berg

**Affiliations:** a Institute of Health and Care Science, Sahlgrenska Academy, University of Gothenburg, Gothenburg, Sweden; b Gothenburg Centre for Person-centred Care, University of Gothenburg, Gothenburg, Sweden; c Department of Obstetrics, Sahlgrenska University Hospital, Gothenburg, Sweden

**Keywords:** Type 1 diabetes mellitus, pregnancy, labour and birth, post-natal care, experiences of care, phenomenology, reflective lifeworld research

## Abstract

Being pregnant, giving birth, and becoming a mother the first months after birth, is for women with type 1 diabetes mellitus (T1DM) a period of difficult challenges. In order to identify their need of support from healthcare, the aim of this study was to describe healthcare during pregnancy, labour, birth, and up to 12 weeks after birth as experienced by Swedish women with T1DM. We used a phenomenological reflective lifeworld research approach, and made 1-2 individual interviews with ten women in late pregnancy and/or 2-3 months after. Transcribed interviews were analysed through focusing on the meanings of the study phenomenon. The results revealed how the diabetes disease, as well as the risks and responsibility that comes with it, become more visible during the period in question, due to a constant monitoring, performed by the woman herself as well as by the healthcare professionals. The essential meaning of the phenomenon is a need to share the burden of risks and responsibilities with healthcare professionals. The complex situation that these women are in, both as experts on their illness and care and in need of care, requires a care that make women feel capable and responsible, but at the same time offers support and relieve them of their responsibility when needed.

## Introduction

Women with type 1 diabetes mellitus (T1DM) face difficult challenges during pregnancy and after birth. Because of their diabetes, there is a higher risk of adverse outcomes for both themselves and the expected and newborn child (Colstrup, Mathiesen, Damm, Jensen, & Ringholm, 2013; Feldman & Brown, 2016; Ringholm, Mathiesen, Kelstrup, & Damm, 2012). Even though the healthcare and the medico-technical equipment to manage the diabetes has improved greatly in the last decades, pregnancy and childbirth are still regarded as a period of increased risks, and for the women it is still a challenge to manage this situation. Therefore, high-quality professional healthcare and support are of great importance for women with T1DM.

Our MoDiab (Motherhood and Diabetes) research group has conducted explorative studies on the situations for women in Sweden having T1DM and being pregnant and in early motherhood. We found that to minimize the increased risks, the women struggled to achieve normoglycaemia 24 h a day. Their main tools to reach this were frequent or continuous glucose monitoring, strict lifestyle habits with diet and physical activities under control, and constant insulin dose adjustments. During pregnancy, they were often filled with worries, pressure to reach optimal glycaemia, and guilt when it was abnormal. (Berg & Honkasalo, 2000, 2005b; Sparud-Lundin & Berg, 2011). In addition to this, they felt that the healthcare both during and after pregnancy primarily focused on the health of the expectant and newborn child, and not on themselves (Sparud-Lundin & Berg, 2011). The first months after birth were a period of extraordinary exposure. The connection to the pregnancy-related professional healthcare usually ended when the baby was born or some weeks after, which contributed to the exposed condition. Breastfeeding was found to be a great challenge, especially during the two first months after birth, a period when the mothers’ glycaemia usually was unstable. Despite this, most of them managed to breastfeed—partly or exclusively—80% at 2 months and 61% at 6 months (Sparud-Lundin, Wennergren, Elfvin, & Berg, 2011). The first months after birth were for the mothers with T1DM a period of high tiredness, higher than for women without diabetes. They had an increased need to organize their daily lives (Berg & Sparud-Lundin, 2012), and their well-being was negatively influenced if breastfeeding affected their diabetes management (Berg, Erlandsson, & Sparud-Lundin, 2012).

## Aim

When it comes to professional healthcare support to women with T1DM in pregnancy, labour, birth and soon after giving birth, there is an evident lack of qualitative studies that consider the whole period from pregnancy through labour, birth, and first months after birth. We, therefore, conducted a study in Sweden to describe the lived experience of healthcare in women with T1DM, during pregnancy, labour, birth, and up to 12 weeks after birth.

## Method

The study was carried out by means of a phenomenological, reflective lifeworld approach (Dahlberg & Dahlberg, 2019b; Dahlberg, Dahlberg, & Nyström, 2008). Important in this approach is the focus on meaning as the essential structure of human experience, and the methodological implications and challenges that follow (Dahlberg & Dahlberg, 2019a, 2019b). With its basis in phenomenological epistemology and lifeworld theory, the reflective lifeworld research approach makes it possible to reflect on tacit meanings of human existence, such as the experience of healthcare. The purpose of reflective lifeworld research is to describe a phenomenon in depth, with the help of a meaning structure that show the essential meanings as well other constituent meanings of the phenomenon. The crucial element in this search for meanings is the researcher’s presence and openness, an attitude referred to as “bridling”. With a bridled attitude, one is obliged to problematize and reflect on taken-for-granted assumptions in order to be present to the phenomenon in question, and let it show itself more fully. The understanding process is slowed down in order to let new and perhaps surprising meanings arise that otherwise might have been clouded by the researcher’s pre-understandings, theories or other established meanings of the phenomenon. The goal of bridling is to reach that presence and attentiveness where one can be open for the new and unexpected (Dahlberg, in press). The bridling process is, however, not something that is performed once and for all, but rather implies an ongoing attitude throughout the process of planning the project, gathering and analysing data, and reporting the study (Dahlberg & Dahlberg, 2019b; Dahlberg et al., 2008).

### Setting and participants

Sampling within a phenomenological approach is concerned with gathering richness and depth in experience (Dahlberg et al., 2008). Two different sampling strategies were used to ensure a varied selection of participants. Swedish-speaking women at a hospital-based secondary antenatal clinic in western Sweden were invited by their diabetes nurse to participate when they were in their third trimester of pregnancy. Eight women accepted, but two later declined to participate, one for private reasons, the other because her baby was born prematurely. In addition, Swedish-speaking pregnant women and new mothers with T1DM were invited to participate through an open invitation in a Facebook group for parents and pregnant women with diabetes. Four women with T1DM accepted participation, of whom two were pregnant and two had recently given birth.

Thus, in total, 10 women participated in the study. All participants lived in western or central Sweden. Some lived in a big city, some in a small city, and some in a rural area. They were between 27 and 37 years, and have lived with diabetes between 14 and 26 years. Nine of them were primiparous, thus being pregnant and giving birth to their first child. One woman had given birth once before. All of them lived with a male partner (Table I).

**Table I.** Characteristics of the study participants

Participant	Age	Diabetes duration, years	Parity after birth	Type of care during pregnancy*	Education	Relationship status	Insulin administration	Interviews, numbers
1	28	17	1	1	Secondary level	Domestic partner	Pump	1
2	32	17	1	1	University	Domestic partner	Pen	2
3	32	18	1	1	Secondary level	Domestic partner	Pen	2
4	26	15	1	3	Secondary level	Domestic partner	Pen	2
5	30	19	1	1	University	Married	Pump	2
6	30	26	1	2	Secondary level	Married	Pump	1
7	34	14	2	1	University	Domestic partner	Pump	2
8	30	21	1	3	University	Married	Pump	1
9	37	25	1	1	University	Married	Pump	1
10	27	17	1	2	Secondary level	Domestic partner	Pump	1

*1 = Care given exclusively at hospital-based antenatal clinic for women with higher risks (secondary level care), by a multidisciplinary team of midwife, obstetrician, diabetologist, diabetes nurse, and dietician.

2 = A combination of hospital-based secondary antenatal care and hospital-based diabetes healthcare.

3 = A combination of primary antenatal care, secondary antenatal care, and diabetes care at hospitals.

In Sweden, there is lack of a national consensus guideline for how to organise perinatal healthcare of women with T1DM. In our study, the women received care according to one of the following three models: 1. Care was given exclusively at a hospital-based antenatal clinic for women with higher risks (secondary level care). The care was conducted by a multidisciplinary team consisting of midwives, obstetricians, diabetologist, diabetes nurses, and dieticians. 2. A combination of hospital-based antenatal care (secondary level care) and hospital-based diabetes healthcare. 3. Combined primary antenatal care, secondary antenatal care, and diabetes care at hospitals (Table I). Following the usual perinatal care programme in Sweden, a follow-up was conducted at the respective antenatal care unit 6 to 12 weeks after birth; thereafter, the women were all enrolled in their regular diabetes care.

The second author of this study is partly employed at one of the antenatal clinics that is included in the study, but has not been involved in the care of the participants. The first author does not have any connection to the clinics in this study.

### Interviews

Five of the women were interviewed once and five were interviewed twice—once between 28 and 36 gestational weeks and once 8 to 12 weeks after birth. The reason for conducting two interviews was to obtain experiences both during pregnancy and after birth, and to provide room for reflection for the interviewee between the two interviews. Because of the different selection strategies, however, four of the women had already given birth at the time of the first interview, and were thus only interviewed once, between 8 and 12 weeks after birth. One of the women whom we planned to interview twice was not reachable at the time for the second interview, and was thus only interviewed once. Thus, in total, 15 in-depth, open interviews were performed (Table I).

The participants chose the place and time for the interviews, which all were conducted by the first author. Nine interviews were conducted in the participants’ homes, four in a room at the university, one at an antenatal clinic and one was conducted by phone due to long distance. To introduce a sense of ease, and to allow for the lived experiences of daily life rather than ready-made ideas, the participants were first asked to describe an ordinary day. Thereafter, questions regarding the study phenomenon were asked, such as “how did you experience healthcare during your pregnancy?” A bridled attitude was used during the interviews, and questions were formulated carefully, focusing on question areas such as the experience of healthcare during pregnancy, labour, and birth, and after birth (Dahlberg et al., 2008). The participants were encouraged to elaborate on experiences and concrete situations from their everyday lives, related to the study phenomenon. Questions were put as openly as possible, and many follow-up questions were asked in order to deepen the description and allow for tacit meanings to become explicit. The follow-up questions served to openly explore the meaning of a statement or experience, instead of prejudging it (Dahlberg & Dahlberg, 2019a). All interviews were audio recorded and lasted between 32 and 98 min (mean 60 min).

### Analysis

The interviews were transcribed verbatim and analysed according to the reflexive lifeworld research approach. Such analysis is characterized by a constant movement between particular meanings in each interview and the interview as a whole, as well as between each interview and all interviews as a whole. The process of analysis went from discovering meaning units to finding patterns of meaning, an essential structure, and finally, variations of the meaning patterns, described below as constituents (Dahlberg & Dahlberg, 2019b; Dahlberg et al., 2008).

Each transcript was first to read repeatedly to attain a sense of what the transcript pointed to as a whole. A closer reading of each transcript was then performed in order to divide it into meaning units that answered to the aim of the study. For the meaning patterns to emerge, we asked questions such as “What is being said?” “How is it said?” “What is the meaning of this?” as well as “Is this the actual meaning being expressed, or does it mean something else?” As a part of the bridling process, we also repeatedly asked ourselves “Why do we understand it like this?” The meaning units where then clustered into temporary patterns of meanings that extended across the interview texts. From this, the essential meaning that characterized the phenomenon was identified. The clustered meaning units were then further synthesized into constituents that elucidated their interrelationships and further clarified the essential meaning and the phenomenon as a whole. The essential meaning together with the constituents forms a meaning structure that illuminates the phenomenon in focus.

As a part of the bridling process, questions were continually asked about whether this was the best way to understand the phenomenon, or whether it should be understood differently. The meaning constituents and the essence were worked out collaboratively by both researchers (HD and MB). Below, under *Results*, we first present the essential meaning of the phenomenon, which serves as a background against which the following meaning constituents can be understood as figures. The constituents are not exclusive, but on the contrary overlap to some extent.

### Ethical considerations

All procedures performed in this study were in accordance with the ethical standards of the institutional research committee and with the 1964 Helsinki declaration and its later amendments (World Medical Association, 2013). Ethical approval was received from the Regional Ethics Board (Diary number: 1155-17). Each participant received both verbal and written information about the study. They were told that the interviews would be recorded, and that they had the right to withdraw from the study at any time. Informed consent was received prior to the interviews.

## Results

The lived experience of having T1DM, being pregnant and giving birth, and of becoming a mother the first months after birth, appears as a period in which the woman to an even higher degree than usual must relate to her diabetes disease and the risks and responsibility that comes with it. The process from pregnancy to after birth is characterized by a constant monitoring, performed by the woman herself as well as by the healthcare professionals. This highlights the diabetes illness, its risks, and the responsibility associated with it, before, during, and after the birth of the child. It gives rise to a wish to share the burden of risks and responsibilities with healthcare professionals, and to get support from them. For this to be possible, the women need to be in a personal and friendly relationship with the healthcare professionals, a relationship that can offer individual and sensitive support, and ease feelings of responsibility and guilt.

The meaning of the study phenomenon is described in five constituents, as follows, and with quotes from the participants labelled as P1 to P10.

### Constant monitoring highlights the illness, the risks, and the responsibility

All women had lived with the diabetes disease for a long time, between 14 and 26 years. Being pregnant was, however, something completely new for all of them except for one, who had given birth once before. The pregnancy changed their understanding of, and way of relating to, their illness. Being pregnant with T1DM implied an increased attention to the diabetes disease, with constant monitoring of blood glucose as well as meticulous control and evaluation of food intake. One woman expressed that whereas before she related to her illness as a slight impairment, like having to wear glasses, she had now come to understand it as critical, since her blood glucose levels would immediately affect her unborn baby.

This unceasing control was described in words such as “hysterical” and “exhausting” and as “a full-time job”. The technological tools that measured the glucose in the body’s interstitial fluid, such as continuous glucose monitors and flash glucose monitor systems, provided the possibility for constant surveillance, which was highly appreciated by the women. At the same time, the medico-technical equipment also called attention to the illness, which made it come into focus *as* illness.

I’m never so much a diabetic, I’m never so ill, as when I am pregnant. How can I say … you embrace your disease much more. It’s not that the illness gets more ill but rather … [that] I have entered a place where it’s my job to keep my blood glucose. That’s my job and it’s a full-time job, sort of … [The illness] is much more present. (P7)

The more gears, the more ill I feel. I get reminded of it all the time when they are there and alerting me, and every time an alarm goes off it’s like; “Hello, you have diabetes!” You have to take this into account … So that’s why these technical gears are really two-sided for me, they help a lot, but they are also like a constant reminder that … hello, you are also sick, don’t forget it. It’s a little bit like it’s there, tapping on your shoulder, all the time. (P9)

The numerous visits to maternity and diabetes care facilities with examinations of different kinds furthermore emphasized the diabetes illness and the increased risks it caused. Every examination was an occasion where the healthcare professionals could deliver bad news.

Well it’s … like a two-edged sword (laughs). To have that much healthcare surrounding you brings to mind … I know that it comes with risks … The last two or three weeks you were there … once a week or once every second week and did CTG to check that the baby was alive and stuff like that. And of course there is an increased risk of them dying in the womb and stuff like that and these risks you carry with you. At other times, I don’t feel ill with my diabetes. But now I feel much more that I have an illness. It’s not a normal pregnancy. (P7)

Being pregnant with diabetes thus implied to constantly monitor oneself, but also being constantly monitored by healthcare. The women appreciated the fact that they and their babies were continuously supervised by healthcare professionals, but the apparent risks that prompted this supervision also served to emphasize their vulnerability as well as their responsibility.

Something happened when it was no longer about my own body, but also about someone else’s body, that I’m responsible for, solely responsible. My husband only sees the belly growing. But how the baby’s heart is developing and things like this, it’s all on me. If my [blood glucose] values are too high, my baby can become ill. That’s why the diabetes was allowed to be more present … I have thought to myself many times, both during pregnancy and now afterwards, like this: why didn’t they try to dissuade me from becoming pregnant? Because this, it doesn’t add up, having diabetes and […] taking care of a small child. (P9)

### Needing professional support to lift the responsibility and ease the guilt

The women were in a situation that was new, and in some ways scary, for them. Their illness and the risks associated with it were suddenly more present, as well as the responsibility that came with it. These circumstances set specific requirements on the healthcare that they received, as well as on the relationships between the women and the healthcare professionals. The healthcare that they received could ease anxiety, but could also make it worse.

The women wanted to be “cared for” by the healthcare professionals. “Being cared for” included many nuances, from receiving professional guidance to feeling supported and taken care of. In order to feel taken care of, the women needed to trust the healthcare professionals, their knowledge and advice. Such trustful relationships had the power to lift the responsibility of the women’s shoulders, or at least make it easier to carry. For example, they wanted to hear that they were doing a good job, that they were doing all that could be expected of them, in order to be able to relax and to let go of anxiety. Such affirmation was crucial, as being pregnant implied an underlying feeling of guilt for putting the child at risk.

It usually feels very nice, because I have very high demands on myself and when I’m there [at the antenatal clinic] I always get confirmation that I’m doing a good job, they tell me to relax. (P1)

On the whole, the women expressed that they had been well supported and encouraged by the healthcare professionals. However, one woman experienced the very opposite during pregnancy, that she was criticized by several doctors when she hadn’t done “well enough” in relation to her blood glucose levels. This left her feeling even more guilty:

You feel bad, like a bad parent, even though you are still not a parent. It’s like … you need to feel you are doing something right if you already have a bad conscience, because you are, you have diabetes, and you think it’s really stupid that you want a child and you have a guilty conscience for it. Even if it doesn’t mean that the child will have anything [any damage], you still have a guilty conscience. And then you need some boost to feel a little ok. But it … doesn’t happen, and instead they reinforce the anxiety that you already have. (P10)

### Needing to be met as a unique person

A need to be met, seen, and understood as a unique person was prominent among the women. They appreciated when the healthcare professionals expressed genuine concern for them, as opposed to “just doing their job”. Such a relationship was described as personal and friendly and was, for example, experienced when the professionals took time to visit the women at the postnatal ward, or expressed joy when seeing the newborn baby. One woman had experienced such a genuine relationship with her diabetes nurse and described it in the following way: “She is someone that I could dare to call almost in the middle of night, if I were to need it.” (P4).

To be met as a person also implied that the healthcare professional met the woman as she was at that moment, and with her special resources and needs, instead of only following the care manuals.

She starts from me and my needs. It’s not so much “this is how you do it” but rather “this is what we can do for you”. And this is the alpha and omega of diabetes care. There’s not a memo that tells you that you should raise this or do that, but “this is how I am”. Since there’s so much self-care, it’s me who best knows my diabetes, while she [the healthcare professional] is someone that I should be able to consult as a professional. (P7)

She said: “I know that I have to be like this with you.” And then it felt like, wow, you know me. It was so awesome that she could put into words and had found who I was in a caring situation. (P9)

To be met in this way, as the person one is, was a prerequisite for feeling cared for, and for wanting to hand oneself over to the healthcare professionals and system. However, this did not imply completely giving up the control or turning into a passive receiver of care. The women knew that they had to manage their diabetes by themselves, but they needed help and support to be able to help themselves. Because diabetes is an illness that is dependent on self-care, it was crucial that the caring relation was a personal relation. One woman explained the difference between being met as a person and being met as an illness, by recounting a meeting with a nurse who was unable to meet her as a person: “She was more like ‘this is the memo, this is what you do’. And I was not helped by that. Because the illness is such an integrated part of my life.” (P7)

The trustful and personal relationship that the women wished for was facilitated by a continuous contact with one and the same healthcare professional, no matter whether midwife, nurse, or doctor. However, this was not always obtained, and sometimes the opposite occurred, that a woman was constantly treated by different healthcare professionals. To have to tell one’s story and explain the same things over and over again, every time one met with a new professional, was annoying, but could also be painful if it was a hard, embarrassing, or traumatic experience to recount.

P6: Until the weekend we had different midwives every day. Then, during the weekend we had the same for four shifts. And that was good … because she knows our history, she knows our difficulties. She knows what was hard for us during the birth and why we so much want to go home … You don’t want to tell all that again. Especially not when it was difficult.

I: Did you have to do that with the others? Tell everything every time?

P6: Yes, and so I started crying every time.

Instead of feeling seen as a person, the women who constantly was cared for by different caregivers felt that they were replaceable, just like any patient. This made it impossible to build a trustful and personal relationship.

### Being lost in the healthcare system

The women’s experience of healthcare was strongly influenced by how the healthcare was organized. As pregnancy, childbirth, and early motherhood were a new situation for them, they needed to know and understand what was happening in care and why. Otherwise, a feeling of being lost was prominent. To feel lost was the opposite of feeling taken care of, and this feeling arose when the information was not clear or concise enough for them to understand the situation. The feeling of being lost was also noticeable when they did not know who was in charge or had an overview of their care, or when the communication between different professionals and wards was not working. The feeling of being lost in healthcare appeared with different nuances during pregnancy, labour, and early after birth. Thus, this constituent is divided into three different parts: pregnancy, labour and birth, and after birth.

#### Being lost during pregnancy

The feeling of being lost during pregnancy was prominent among the women who received care at different units, such as the diabetes care unit and at primary and/or hospital-based antenatal care facilities. They often did not know who was in control or to whom they should ask their questions. There was also a recurrent feeling that information was missed and that check-ups were missed or done twice. This resulted in insecurity and mistrust, and these women felt lost in a poorly organized and confusing care apparatus. One woman who went to three different healthcare organizations during pregnancy felt that there was no one in charge of her care, and the feeling of being lost was overwhelming. She felt stressed, during pregnancy, about how to manage breastfeeding with her diabetes. When she raised this question with the midwives at the primary antenatal clinic, they were surprised that she had not got this information at the secondary antenatal clinic, and told her that they would probably talk to her soon. But at the secondary antenatal clinic, they never talked about breastfeeding. Shortly after birth, she stopped breastfeeding because of difficulties with maintaining her blood glucose at an even level while breastfeeding.

The women who went to different care facilities also sometimes got conflicting advice. For example, one woman, who met several different diabetes doctors during her pregnancy, had the experience that they overtly questioned each other’s’ advice, which made her feel insecure and mistrust the doctor’s knowledge altogether.

The feeling of being lost also arose when it was not clear what the women were doing in healthcare or why. One woman described this feeling as being “thrown into a care machine”, where she had no real overview, and where things were not explained to her:

Like these CTG curves that I have tried the second week in a row to ask: but why do I do them so often? What do you want to see here compared to someone who doesn’t have diabetes? Obviously they see something, since they find that they are fine. But for me, who only, I only see my baby’s pulse and then I see some kind of number that measures my contractions, but I have no idea what it means. Sometimes it says 11 and then it says 40, and then I’m supposed to press a button when I feel a kick … It’s nice to know that everything’s fine but I don’t understand. (P4)

In contrast to this, the women who received antenatal care by a multidisciplinary team at a hospital-based clinic described a feeling of being taken care of. That the healthcare professionals—midwife, diabetes nurse, and doctor—worked as a team was appreciated and included that unanimous information was given and that the different professionals complemented each other in a well-organized way.

#### Being lost during labour and birth

The needs of being taken care of and knowing what is happening were heightened during labour and birth, as was the feeling of being lost. One of the reasons for this was that giving birth appeared not only as a new and frightening experience, but also as an existential moment of life and death. There were several examples during labour and birth when the women did not know what was happening or why, and where they were given divergent or insufficient information. This gave rise to anxiety and feelings of abandonment and mistrust. The existential significance inherent in the situation and the feeling of being lost was moreover amplified when it appeared as though the healthcare professionals did not know how to handle their diabetes, and the women were left to manage it on their own. One woman described the precarious situation of giving birth and suffering from diabetes in the following way: “It’s like jumping free fall, you can only hope that you get down alive. That your parachute works.” (P6). Another woman, who wanted help from the professionals to manage her diabetes when giving birth, felt that she could not trust them. This left her feeling insecure, abandoned, and frightened, and at times afraid of dying. The fact that she had to give birth and at the same time keep track of her blood glucose levels felt bizarre to her:

I felt lonely. In a situation like that I want to be able to rely on the carers and put them in charge, like I do what you say. If you tell me to push I will push, if you tell me to take insulin I will. But when it came to the insulin for the diabetes, I felt that I couldn’t trust them. The other things I trusted. But if they tell me “take one and a half units”, and I can see that I already have two units active in my body, then I will not take one and a half units more. So I felt like I couldn’t trust them, and then, of course, I felt lonely. It’s hard when you can’t trust the healthcare staff. It does something to you, because you’re already in a vulnerable position and then you just want to be able to let go of control … And I couldn’t […] To try to be there mentally, with your diabetes, at the same time as I have contractions and am inhaling nitrous oxide … it doesn’t add up. Then all responsibility rests on me and partly on my husband. You hadn’t done that in any other circumstance, like: Yeah well you’re going to have surgery but you have to manage the narcosis by yourself. (P9)

Some of the women expressed how they wanted to trust their healthcare professionals, and hand themselves over, but felt that they could not when it came to diabetes care. They felt that they were given poor advice about their blood glucose levels and that the healthcare professionals acted on knowledge they had read in dated memos. Others felt that it was not a problem to manage their diabetes on their own, or together with their partner, when giving birth, since they were used to it, but were annoyed by having to answer to professionals who insisted on, for example, checking their blood glucose manually, through a blood sample, even though they had continuous supervision of blood glucose levels through their monitors.

The woman who had given birth once before differed from the others. She and her partner had also been thoroughly prepared by the diabetes nurse on several occasions, who told them what to do and what to demand. She was thus well prepared and felt competent in her knowledge, and because of this she felt that she could engage in a partnership with the healthcare professionals. Instead of feeling lost in the situation, she felt in control and she experienced support from the professionals: “They saw me and they listened to me and my needs. And they were there with me, informed me, and told me what happened. So that I felt safe. I knew the next step all the time, what will happen and where I am and such.” (P7)

The other women, who did not feel prepared and did not have this confidence as a platform, were not able to engage in such partnership with the professionals.

#### Being lost after birth

Being lost was also noticeable in the women’s experiences after giving birth, especially among those women whose babies required care at a special neonatal care ward. As the co-care rooms were too few, some of the mothers were co-cared with the baby, and some not. The mothers whose babies required neonatal care expressed feelings of being excluded from care, as the neonatal care ward focused solely on the baby, not on the mother. For example, no food or drinks were provided to the mothers, which made it hard to manage diabetes at the neonatal ward. One woman needed something to eat or drink to stabilize her blood glucose when she was at the neonatal ward with her baby. She could not get it there, and she was not allowed to get it from the maternity ward, since she had already been discharged from there. She felt insecure and vulnerable in her situation as a new mother, and at the same struggling with her diabetes disease, which she could not recognize. The absence of support from the healthcare professionals left her feeling abandoned and alone.

Communication between the postnatal ward and neonatal ward was deficient or non-existent. Sometimes mothers were given divergent information from the two different wards about, for example, breastfeeding, and sometimes the mothers missed information on breastfeeding altogether, since they were moving between different wards. This influenced later struggling to breastfeed while at the same time managing their diabetes. One woman, who stayed at the postnatal ward separated from her baby while he was treated at the neonatal ward, described how she, besides missing out information, had a traumatic experience of not being able to connect to her son until six or seven weeks after birth:

When we came to the neonatal ward temporarily, it felt like I was watching someone else’s kid while they were out for dinner. I lay there watching him, like, it is a sweet kid but it’s not mine, sort of. Then when we came home … it was hard for him to eat and so he was tubed (sighs). And it took a long time before I could breastfeed, and I was pumping to get it started. And I was extremely stressed because he wasn’t gaining weight, and I was supposed to solve that with breastfeeding, which wasn’t working … And I couldn’t see him as mine. I feel that I would have needed to stay with him 24/7, like at a normal birth. To be able to bond with him. (P8)

A feeling of being lost was present also in the first weeks after birth, after returning home. The women experienced that there was a gap in their care and in the healthcare professionals’ support. From being in close and regular contact with the antenatal care, with frequent check-ups, they experienced that they suddenly did not know who to turn to when they had trouble or needed support. This gap was unfortunate, as they in this early stage of motherhood were in a challenging and sometimes difficult time. Their blood glucose levels often fluctuated rapidly when breastfeeding, and combined with interrupted sleep, it caused tiredness. Besides the physical exhaustion, there were sometimes also emotional difficulties in trying to deal with two very demanding factors needing constant attention: a new-born baby and their own fluctuating blood glucose levels:

It’s so tough, because I have all the symptoms that come with low blood glucose—I’m trembling and weak and a little dizzy, and at the same time taking care of the needs of a small child. Then it’s also emotionally hard. I get angry because I feel that it’s so hard to have diabetes and at the same time … I have never been so angry at the fact that I have diabetes as since he came into my life. Because it doesn’t fit (laughs). It is disturbing. It disturbs my focus on him. I have to prioritize between two things which I cannot prioritize between. (9)

The women described how they had to develop different strategies to get through this challenging time of early motherhood. Some of them were in contact with their diabetes nurse at the antenatal care centre, with whom they had developed a close contact. Others managed by themselves through trial and error. One woman who did not have any support at all from the healthcare service, and who experienced difficulties trying to breastfeed and manage her diabetes at the same time, eventually stopped breastfeeding.

### Being shut out by medical blinkers

The women’s overall experience of healthcare during pregnancy and birth, and after birth, was that of being seen and met as persons. However, several descriptions were given of when healthcare professionals seemed to remain within their medical perspective, and because of this were unable to see the situation from the perspective of the woman. The difficulty of seeing outside one’s medical perspective can be understood as “medical blinkers”, that is, the medical perspective acts as blinkers that hinder the healthcare professional from seeing the situation from the patient’s point of view. In these descriptions, healthcare professionals communicated and acted on medical information, but did not understand how this information was understood by the patients, and how to transfer this to the patient in a, for her, comprehensive way.

One such situation was described by a woman who had an ultrasound examination in early pregnancy. The doctor performing it explained to her that the foetus’s heart could not be seen. The woman, who had suffered from bleedings during her pregnancy, immediately thought that the foetus was dead, but was too shocked to speak. After a while the doctor continued: “But the brain looks fine.” By herself, the woman then concluded that if the foetus has brain activity, then it must be alive, but she felt speechless from the experience and never asked the doctor if this was the case. The reason for this communication breakdown seems to be that the doctor remained within her medical point of view, which made her unable to understand the situation from the patient’s point of view. The fact that the heart of the foetus was not visible was perhaps due to the foetus’s position in the uterus at the time of the examination, but as this was not explained to the woman, she instead sensed a possible tragedy. The gap between the experienced and all-knowing expert—the healthcare professional—and the inexperienced and vulnerable patient, was then widened.

Another example of when healthcare professionals were blinded by medical blinkers was when the doctor during an ultrasound examination presented the estimated weight of the foetus to the woman in percentages. For the doctor, this was self-evident and had meaning, but the woman who was examined could not relate to it:

The first time I did an ultrasound I got [the information]: “plus 11 per cent”. OK? What’s that, then? I only hear a “plus”, and I think that plus is really bad, because I will have really big children. (P4)

The medical blinkers were also apparent when healthcare professionals only met and treated what fell within the boundaries of medicine, but failed to understand the perspective of the woman. This occurred, for example, when the staff focused only on medical information such as blood glucose levels or foetal growth, while a woman’s feelings and worries connected to being pregnant and having diabetes could be dismissed as “normal” or remitted to a specialist in mental ill health. The medical blinkers then shut out the patient as a person, which is exemplified in the following quote:

Even though the diabetes nurse and the midwife said: “Your blood glucose values are fantastic.” Yes, they are, but the feelings around it … I would have wanted more support there. It was very much like: “Everything is normal. If you are worried, you have to go to another care facility [X] where they have a psychologist.” So that either it’s normal, or otherwise, I have to go somewhere else. That doesn’t really make me want to talk to her again about this feeling. But I’m still worried and I still feel lonely. (P9)

This example shows the discrepancy between the woman’s and the professional’s perspectives. From the medical perspective, the situation could be assessed as normal, and thus it was not necessary to meet or care for the woman’s worries. Should the anxiety exceed certain limits, and be considered “abnormal”, it could be dealt with, but then the woman had to be transferred to another professional expert at another department. This meant that the woman was not being met as she wanted at that moment—with her worries.

## Discussion

The aim of the present study was to describe women’s lived experiences of healthcare during pregnancy, labour, birth, and up to 12 weeks after birth when having T1DM. We found that the diabetes illness and its accompanying risks were more visible during this period, and that the women had a need to share the burden of risks and responsibility with healthcare professionals. For this to be possible, they needed a trustful and personal relationship with them. There was a need to be cared for, implying both receiving professional guidance, getting help to help oneself, and being taken care of. In order to not feel lost, there was also a need to know and understand what was happening in care and why, to know who among the healthcare professionals was in charge and of what, and to sense that the healthcare professionals had control over the situation.

Overall and mostly, the participating women were satisfied with the healthcare received. This included that they had been met as persons, had received support, and could share their responsibility with the professionals. However, there were also occasions when women had not been met as persons, when they did not feel supported, and when they felt lost in a badly organized care apparatus. As, for us, the driving force for doing research within the healthcare arena is to contribute to developing better healthcare, in the following we highlight and discuss some such outstanding parts, which are important to know for healthcare professionals caring for women with T1DM during this important episode of life, and to focus on in future endeavours of healthcare improvements.

### A need to be met as a unique person, and to get support to ease the responsibility and guilt

The heightened responsibility, worries, and self-blame in pregnant women with T1DM have been shown previously (Berg & Honkasalo, 2000). In our present study, we found that the women’s feelings of responsibility and guilt were clearly connected to the constant monitoring performed by the women themselves as well as by the healthcare professionals, and which highlighted the illness. The medico-technical equipment available for this supervision was both a blessing and a curse. It made it easier for the women to control and manage their illness, and at the same time it also constantly reminded them that they were ill, that their illness constituted a risk for their child, and that they could be blamed if anything went wrong. The double-sided nature of advanced medical equipment as well as frequent healthcare check-ups—their advantages as well as their disadvantages—must thus be considered in the healthcare for these women.

A systematic review of research in women with T1DM found that the perception of risk because of having diabetes seems to escalate during the transition period to motherhood, and that the medical care seemed to enhance this escalation (Rasmussen et al., 2013). The results in our present study confirm this, but more importantly, it also points to the form of care these women require to ease their responsibility and their guilt. The women’s need of being cared for requires not only having trust in the professional expertise and the organization of care but also being met at a personal level, being seen as who they are, with their specific needs and resources.

Several of the findings in the present study confirm what has been found earlier, such as that the healthcare professional’s attitudes impact women with T1DM in the episodes of pregnancy, labour, and early motherhood, and that women need supportive healthcare to handle the demanding situation (Berg, 2005a; Berg & Hotikasalo, 2000; Berg & Sparud-Lundin, 2009), as well as that the healthcare professionals, through unwise behaviour, sometimes increase worries and stress (Berg & Sparud-Lundin, 2009). An interview study in England with pregnant women with pre-existing diabetes found that they felt the relationships with the professionals were dominated by advice, judgement, and control rather than by dialogue, and with a focus on blood glucose levels rather than on the women as whole persons and their situation of being pregnant with diabetes (Stenhouse, Letherby, & Stephen, 2013). Our present study contradicts this to some extent, as the overall feeling among the women was that of being supported, encouraged, and met as persons by healthcare professionals.

### Being lost in healthcare

Despite the general experience of being in a personal, supportive, and friendly relationship with healthcare professionals, there were also several examples of feeling lost, including feelings of abandonment and mistrust. These feelings arose for different reasons, one of which was related to poor, fragmented healthcare organization. Being lost could also appear when the women did not know what was happening, thus, when they got insufficient information. To make women feel at home in the healthcare system rather than lost, they thus need clear and concise information regarding what they are doing in healthcare and why, in a language that is not filled with medical jargon but that is meaningful for them.

Feeling lost could also be due to the detrimental effect of a disconnected care organization, where the women with T1DM visited several different clinics during their pregnancy. This was already found in our earlier study a decade ago (Berg & Sparud-Lundin, 2009). Incoherent care was in that study expressed in the form of insufficient communication, as well as unclear distribution of responsibility between the different healthcare providers. This forced the women to act as messengers between these different healthcare actors. Our current study shows that this fragmented care organization is still a problem, and it shows how the poor organization contributed to a feeling of being lost, which gave rise to feelings of mistrust and abandonment and of not being cared for. The feeling of being lost was especially salient during labour and birth, which could be attributed to a lack of knowledge of how to manage diabetes, and a noticeable lack of professional resources at the wards. The women giving birth met with many different professionals, and the lack of information left them feeling abandoned. The feeling of mistrust in the professionals’ knowledge of diabetes probably negatively influenced the physiological labour and birth process, which has been shown to be very sensitive to stress (Uvnäs Moberg, 2014)

There were examples of feeling lost right after birth, as well. This feeling was dominated by the lack of communication between postnatal and neonatal wards. The first weeks after birth were in turn characterized by a disconnectedness from the healthcare service.

### Healthcare professionals with medical blinkers

One reason for the women not being met and seen as persons was the healthcare professionals being blinded by “medical blinkers” and thus unable to see the situation from the woman’s perspective. This led to a failure to meet, see, and talk with the woman in the way that she needed. The example of the woman who, during the ultrasound, was informed that the doctor could not find the foetal heart illustrates this. The doctor was so absorbed in her own medical perspective, including the task of identifying all the different organs during the ultrasound, that she forgot to communicate in a professional and human way with the woman. The reason why a doctor speaks about a missing heart without realizing how this is conceived by the patient is probably not malevolence, but more likely unawareness and a difficulty to see beyond one’s own perspective. One way to look at this communication breakdown is by describing the two different “voices” employed by patients and healthcare professionals in situations like this. Mishler (1984), and later Barry et al. (2001) have described how healthcare professionals sometimes use “the voice of medicine” while patients speak with “the voice of the lifeworld”. So while patients speak in an everyday language about their concerns, where their concerns and symptoms are tied up with their existence, healthcare professionals stay rigidly inside a biomedical format, a format that is de-contextualized and impersonal. The result is that the patient is more or less reduced to a disease, and not met as a unique person. Mishler’s understanding of these two voices is appropriate also for the blinkered vision that is evident in our result. However, Mishler’s understanding of the phenomenological concept of the lifeworld is somewhat simplified, and a more profound understanding of the lifeworld, as a meaningful, interconnected world, might also give more suggestions as to what it is to meet a patient as a unique person, as a human being focusing on health rather than disease (Dahlberg, Todres, & Galvin, 2009; Todres, Galvin, & Dahberg, 2007).

Finally, the results of our study give us reason to discuss the very notion of person-centred care. One of the driving forces behind the change to a more person-centred care is the motivation to turn the passive patient, thought of as a mere recipient of care, into an active one, through partnership and empowerment (Ekman et al., 2011; McCormack & McCance, 2006). The notion of partnership thus implies that the patient is not only involved in his or her own care, but also thought of as an autonomous person who should be given the responsibility to make his/her own choices. Our present study complicates this understanding of person centredness somewhat. It is a somewhat paradoxical situation of being pregnant, giving birth, and becoming a mother when having T1DM. The women are experts on living with their own illness, while at the same time being in need of specific professional healthcare, and thus they have to give up, or lose, some of their control. At the same time as they are used to handling their diabetes, they are in a vulnerable position where the illness, and what it implicates in terms of risks and responsibility for the child, comes to the foreground. Moreover, the diabetes can show itself in new ways that they do not recognize. In this study, the women give voice to a need to share and be relieved of the burden of responsibility.

A study on women with diabetes before pregnancy has pointed to the importance of involving women in their own care, and of giving them the power to make their own choices as well as to take responsibility for their actions, when they wish to do so (Stenhouse et al., 2013). If the care relationship is a one-way consultation, dominated by judgement and control, as was found by Stenhouse et al. (2013), the first step is, of course, to involve women in their own care. However, this does not and should not imply that women are left with the responsibility to decide for their own care, or that they cannot be relieved of some control when desired. One of our previous studies has suggested the need to clarify responsibility between the women and the healthcare professionals, for example, during childbirth, since women can have a wish both to stay in control and to be relieved of control (Berg & Sparud-Lundin, 2009). In the present study, the women expressed a need to be cared for, which implied both professional guidance to help oneself and a need to be taken care of.

Our current study thus points to the complex dynamic between woman and healthcare professional that offers the possibility to be relieved of responsibility, but that at the same time strengthens the woman to care for herself and her baby. It would thus be too simple to state that women either are in control or else they give up control to healthcare professionals—that they are either active or passive. What our study shows is the need of these women to be in a personal relationship where responsibility can be shared with healthcare professionals, and where one is both in control and at the same time taken care of. The feeling of being close to the healthcare professional, in a personal and friendly relationship where one is seen as the person one is, formed the foundation for trust, strength, and capability to care for oneself and one’s baby.

Furthermore, our study suggests that the possibility of engaging in a partnership with healthcare professionals requires that the patients feel themselves to be in control of the situation and confident in their own knowledge. On occasions where one feels lost or vulnerable, it is not so easy to demand one’s rights or to engage in a partnership. It thus seems that the notion of partnership should be discussed more. What is needed for patients to be able to form a partnership with healthcare professionals, in terms of trust (both in oneself and the professional), knowledge, and feelings of safety?

## Conclusion

Our present study shows that although the healthcare and the medico-technical equipment to manage the diabetes has improved greatly, the period of pregnancy, childbirth, and early post-partum is still a great challenge to manage for women with T1DM. Refined techniques to control the condition of the foetus and newborn, and the mother’s blood glucose and insulin dosages, cannot replace the personal interaction with healthcare professionals. These techniques, as we have shown, furthermore serve to emphasize the illness and the risks. Moreover, the complex situation that these women are in, both as experts on their illness and care and in need of care, requires a care that make women feel capable and responsible, but at the same time offers support and relieve them of their responsibility when needed. It requires a care that at the same time put women in control and relieves them of control.

## Clinical implications of this study

Our study points to several areas where healthcare for pregnant women and mothers with T1DM could be improved:

- Clear and concise information regarding the purpose and planning of healthcare visits and examinations should be given in an understandable language free from “medical jargon”.
- Different professionals working with the same patient should work in collaboration in order to e.g., be able to plan the caring process from pregnancy to after birth, to give unanimous and coherent information, and to make clear for the patient who is in charge, and of what.
- The lack of knowledge among some healthcare providers of how to handle diabetes and especially issues around insulin doses and food could be solved by having a diabetes specialist on-call, e.g., a diabetes nurse with this competence, available, e.g.,, on labour and post-natal wards.
- The disadvantages of advanced medical equipment and frequent healthcare check-ups, such as the highlighting of illness and risk, should be considered by healthcare providers.
